# Detection of genes mutations in cerebrospinal fluid circulating tumor DNA from neoplastic meningitis patients using next generation sequencing

**DOI:** 10.1186/s12885-020-07172-x

**Published:** 2020-07-25

**Authors:** Yue Zhao, Jun Ying He, Jun Zhao Cui, Zi-Qi Meng, Yue Li Zou, Xiao Su Guo, Xin Chen, Xueliang Wang, Li-Tian Yan, Wei Xin Han, Chunyan Li, Li Guo, Hui Bu

**Affiliations:** 1 Department of Neurology, The Second Hospital of Hebei Medical University, 215 Heping West Road, Shijiazhuang, Hebei China; 2 Wenzhou Medical University, Wenzhou, China; 3 San Valley Biotechnology Incorporated, Beijing, China

**Keywords:** Neoplastic meningitis, Cerebrospinal fluid ctDNA, Next generation sequencing, Cancer-associated genes mutations, PI3K-Akt pathway

## Abstract

**Background:**

This study profiled the somatic genes mutations and the copy number variations (CNVs) in cerebrospinal fluid (CSF)-circulating tumor DNA (ctDNA) from patients with neoplastic meningitis (NM).

**Methods:**

A total of 62 CSF ctDNA samples were collected from 58 NM patients for the next generation sequencing. The data were bioinformatically analyzed by (Database for Annotation, Visualization and Integrated Discovery) DAVID software.

**Results:**

The most common mutated gene was *TP53* (54/62; 87.10%), followed by *EGFR* (44/62; 70.97%), *PTEN* (39/62; 62.90%), *CDKN2A* (32/62; 51.61%), *APC* (27/62: 43.55%), *TET2* (27/62; 43.55%), *GNAQ* (18/62; 29.03%), *NOTCH1* (17/62; 27.42%), *VHL* (17/62; 27.42%), *FLT3* (16/62; 25.81%), *PTCH1* (15/62; 24.19%), *BRCA2* (13/62; 20.97%), *KDR* (10/62; 16.13%), *KIT* (9/62; 14.52%), *MLH1* (9/62; 14.52%), *ATM* (8/62; 12.90%), *CBL* (8/62; 12.90%), and *DNMT3A* (7/62; 11.29%). The mutated genes were enriched in the PI3K-Akt signaling pathway by the KEGG pathway analysis. Furthermore, the CNVs of these genes were also identified in these 62 samples. The mutated genes in CSF samples receiving intrathecal chemotherapy and systemic therapy were enriched in the ERK1/2 signaling pathway.

**Conclusions:**

This study identified genes mutations in all CSF ctDNA samples, indicating that these mutated genes may be acted as a kind of biomarker for diagnosis of NM, and these mutated genes may affect meningeal metastasis through PI3K-Akt signaling pathway.

## Background

Neoplastic meningitis (NM) refers to the dissemination of malignant cells to the leptomeninges, and is associated with very poor survival of patients [1]. The primary cancers are mostly lung and breast cancers or brain tumors, such as medulloblastoma. In clinic, early NM detection and timely treatment could significantly impact the outcome of patients. However, the present diagnosis is primarily based on the clinical signs and symptoms plus cerebrospinal fluid (CSF) cytology and neuroimaging [2]. Furthermore, although the detection of tumor cells in the CSF is the key to make NM diagnosis, the CSF cytology may not be reliable due to insufficient sensitivity and specificity. Thus, the detection of cell-free circulating tumor DNA (ctDNA) could be another diagnostic strategy [3–5]. This can detect cancer-associated genes and gene alterations in the plasma or CSF to monitor the tumor progression and/or treatment responses [3–5]. In patients with brain tumor, the plasma ctDNA analysis has revealed either its absence, or extremely low levels [5, 6]. Fortunately, CSF ctDNA has been well demonstrated to be present and even abundant in brain tumor patients [6, 7]. In order to better understand characterization of ctDNA in CSF of NM patients, the detection of mutated genes in the CSF could help medical oncologists identify the primary tumor and make effective treatment options for patients. Therefore, the present study aimed to investigate the gene mutations in the CSF ctDNA samples obtained from NM patients using the cutting-edge technique of next generation sequencing (NGS). This approach can help to characterize NM **genetic profiles** and profile of gene mutations, which can thereby be potentially applied for molecularly targeted therapy. Towards this end, a recent study [8] genetically profiled the CSF ctDNA obtained from NM patients with epidermal growth factor receptor (*EGFR*)-mutant non-small cell lung cancer (NSCLC). Therefore, characterization of gene mutations in CSF ctDNA samples can provide valuable clinical guidance for precision medicine.

## Methods

### Study population and samples

In the present study, a total of 58 patients with NM, who received lumbar puncture for the CSF cytology examination between October 2014 and June 2018 in the Department of Neurology, The Second Hospital of Hebei Medical University (Hebei, China), were enrolled. The diagnosis of these 58 NM patients was entirely according to the clinical signs and symptoms, positive CSF cytology, and/or neuroimaging findings, such as contrast-enhanced brain magnetic resonance imaging (MRI) or computed tomography (CT), and the results from the CSF ctDNA. The NM clinical signs and symptoms included headache, nausea, vomiting, convulsion, lower back pain, cranial nerve paralysis, paresthesia, gait disturbances, vertigo and defects. The positive CSF cytology was defined by the distinctive pattern of the neoplastic cell morphology. That is, cells that had an irregular size and shape, and contained large and polymorphic nuclei with a lobulated state and malformed buds. The chromatin size increased with the basophilic coarse particles, and the mitotic activity was enhanced with aberrant mitosis. The nuclear membrane was usually thickened with a saw-tooth-shaped and wear edge. In addition, the positive neuroimaging revealed the presence of leptomeningeal enhancement. The clinical data are presented in Table 1.

**Table 1:** Characteristics of 58 NM patients

Clinical characteristics	# of patients (%)
Gender	
Male	27 (46.6)
Female	31 (53.4)
Age (year)	
Median (range)	55 (23–77)
Primary cancer	
Lung cancer	42 (72.4)
Gastric cancer	4 (6.9)
Breast cancer	3 (5.2)
Rectal cancer	2 (3.4)
Prostatic cancer	1 (1.7)
Parotid carcinoma	1 (1.7)
Lymphoma	1 (1.7)
Glioblastoma	1 (1.7)
Unknown malignancy	3 (5.2)
First symptom with NM	10 (17.2)
Diagnostic basis	
CSF cytology	44 (75.9)
Neuroimaging findings	8 (13.8)
CSF ctDNA	6 (10.3)
Treatment regimen (CSF samples)	
Intrathecal chemotherapy and systemic therapy	30 (48.4)
Intrathecal chemotherapy without systemic therapy	11 (17.7)
Systemic therapy without intrathecal chemotherapy No therapy	12 (19.4) 9 (28.5)
Hydrocephalus	
Yes	5 (8.6)
No	53 (91.4)

The present study was approved by the Ethics Committee of the Second Hospital of Hebei Medical University (Hebei, China), and a written informed consent was obtained from each patient or their legal surrogates.

### CSF cytology examination

About 0.3 ml CSF was centrifuged at 750 r/min for 4 min (Therm-4, Shandon Cytospin, US). After naturally drying on the slide, the deposit was dyed with May–Grünwald–Giemsa liquid (Yucai, Beijing, China) and Alcian blue staining (Yucai, Beijing, China), according to the manufacturer’s protocol, and observed under a light microscope (OLYMPUS-BX41, Japan). The determination of the positive result was as follows: cells that had an irregular size and shape, and contained large and polymorphic nuclei with a lobulated state and malformed buds. The chromatin size increased with the basophilic coarse particles, and the mitotic activity was enhanced with aberrant mitosis. The nuclear membrane was usually thickened with a saw-tooth-shaped and wear edge. Analysis was done using a cell medical image analysis system (MCDS-20, Chongqing, China).

### Next-generation sequencing

#### CSF samples and processing

The CSF samples were collected from each patient and placed into EDTA tubes, according to our hospital routine protocols. Then, these were centrifuged for five minutes at 1000 *g*. The pellet was stored at − 20 °C, while the supernatant was centrifuged at 10,000 *g* for an additional 30 min, according to a previous study [9]. The supernatant was transferred into pre-labeled cryotubes and stored at − 80 °C. Next, the ctDNA was extracted from at least 5 mL of the CSF supernatant using a QIAamp Circulating Nucleic Acid kit (QIAGEN), according kit instructions, and the ctDNA was quantified using a Qubit2.1 Fluorometer and Qubit dsDNA HS Assay kit (Life Technologies, Carlsbad, CA, USA).

#### Preparation of the ctDNA library and the next generation DNA sequencing

The ctDNA samples were subjected to preparation of the Ion Proton library and DNA sequencing, according to the methodologies from previous studies [10–12]. Briefly, for each sample, an adapter-ligated library was generated using the Ion AmpliSeq Library Kit 2.0 (Life Technologies). That is, the pooled amplicons made from 10 to 20 ng of ctDNA samples were end-repaired and ligated to Ion Adapters X and P1, and purified using AMPure beads (Beckman Coulter, Brea, USA) to obtain the adapter-ligated products, followed by nick-translation and PCR-amplification for a total of five cycles. Then, the products were subjected to analysis using the Agilent 2100 Bioanalyzer and Agilent Bioanalyzer DNA High-Sensitivity LabChip (Agilent Technologies) to determine the concentration and size of the library, and sample emulsion PCR and emulsion breaking using the Ion OneTouch™ 2 system (Life Technologies) with the Ion PI Template OT2 200 Kit v3 (Life Technologies), according to manufacturer’s instructions. Afterwards, the Ion Sphere Particles (ISPs) were recovered, and the template-positive ISPs were enriched with Dynabeads MyOne Streptavidin C1 beads (Life Technologies) on the Ion One Touch ES (Enrichment System, Life Technologies) and confirmed using the Qubit 2.0 Fluorometer (Life Technologies).

The barcoded samples were sequenced using the Ion Proton System with Ion PI v2 Chips (Life Technologies) for 100 cycles, while the Ion PI Sequencing 200 Kit v3 (Life Technologies) was used for the sequencing reactions. Then, the SV-OCP143-ctDNA panel (San Valley Biotech Inc., Beijing, China) was used to detect the somatic mutations of 143 cancer-related genes. Since the CSF ctDNA samples contained short DNA fragments, the amplicons in the panel were specially designed for the efficient amplification of ctDNA, and the total read counts were more than 25 million to ensure an average base coverage depth over 10,000 folds. In addition, strict quality control criteria were used to ensure that the average uniformity of the base coverage is no less than 95.5% for the reliability of the DNA sequencing and mutation detection.

#### Processing and analysis of DNA sequencing data

The raw DNA sequencing data were processed and analyzed using the Ion Proton platform-specific pipeline (Torrent Suite v5.0) with a specific plug-in (Variant Caller v5.0), which included the readouts of the raw DNA sequences, the trimming of the adapter sequences, and the filtering and removal of poor signal sequences according to previous studies [10–12]. These three filtering steps were applied to eliminate the erroneous base calling, and the final variant calling was generated. That is, the first step evaluated the DNA sequences using the following criteria: the average total coverage depth is > 10,000, each variant coverage is > 10, the variant frequency for each sample is > 0.1%, and the *P-*value is < 0.01. The second step was to eliminate ant potential DNA strand-specific errors after visual examination of the gene mutations using the Integrative Genomics Viewer (IGV; http//www.broadinstitute.org/igv) or Samtools (http://samtools.sourceforge.net) software. For the third step, the total amplicon read counts from the Coverage Analysis Plugin were utilized to identify the copy number variants (CNVs). Then, the read counts per amplicon of each sample was normalized to the total number of reads for a given sample, and divided by normalized counts from a composite normal male genomic DNA sample, which yielded a copy number ratio after correcting for GC content. The gene-level copy number was estimated through the determination of the coverage-weighted mean of the GC-corrected per-probe ratio, which was corrected with the expected error, according to the probe-to-probe variance [13]. Afterwards, genes with a copy number of < 1 or > 4 were regarded as loss or gain, respectively.

### *EGFR* mutations in lung cancer tissues

Information of *EGFR* mutations in lung cancer tissues were obtained from patient’s medical record. Lung cancer tissues of most patients were sequenced before this study by various methods.

### Functional annotation and pathway enrichment analysis

Next, the mutated genes were bioinformatically analyzed using the gene ontology (GO) terms [14] and the Kyoto Encyclopedia of Genes and Genomics (KEGG) pathway enrichment analysis with the Database for Annotation, Visualization, and Integrated Discovery (DAVID; v.6.8, https://david.ncifcrf.gov/tools.jsp). Then, the data were clasisified as the functional annotation and the KEGG pathway enrichment. A *P*-value of < 0.05 was set to be statistically significant.

### Statistical analysis

The statistical significance in gene mutation frequency between the two groups was analyzed using Fisher exact test. Pearson correlation analysis was used for correlation analysis. Nonparametric test was used for two independent samples that did not meet the normal distribution. Statistical analyses were performed using SPSS version 19, and a two tailed *P*-value of < 0.05 was considered statistically significant.

## Results

### Patient characteristics

Clinical characteristics are shown in Table 1. For NM patients with lung cancer, the majority of these patients had lung adenocarcinoma (27/42, 64.3%), while of 55 patients had known primary malignancies and 10 patients (18.2%) had NM as the first clinical manifestation. These patients were followed up for two month or longer.

A total of 62 CSF samples were collected from these 58 NM patients, in which three CSF samples were collected from a single patient, while two CSF samples were collected from other two patients at distinct time points. Furthermore, among the 62 CSF samples, 30 CSF samples were collected from 28 patients who received intrathecal chemotherapy and systemic radiotherapy, chemotherapy, and/or molecule-targeted therapy, 11 CSF samples were obtained from 11 patients who received intrathecal chemotherapy, and 12 CSF samples were obtained from 12 patients who received systemic therapy. The remaining nine CSF samples were collected from nine patients who did not receive any anticancer therapy.

### Cancer-associated genes mutations in the 62 CSF specimens, regardless of the origin of the primary cancer and the mutated genes functional enrichment analysis

**Fig. 1:**
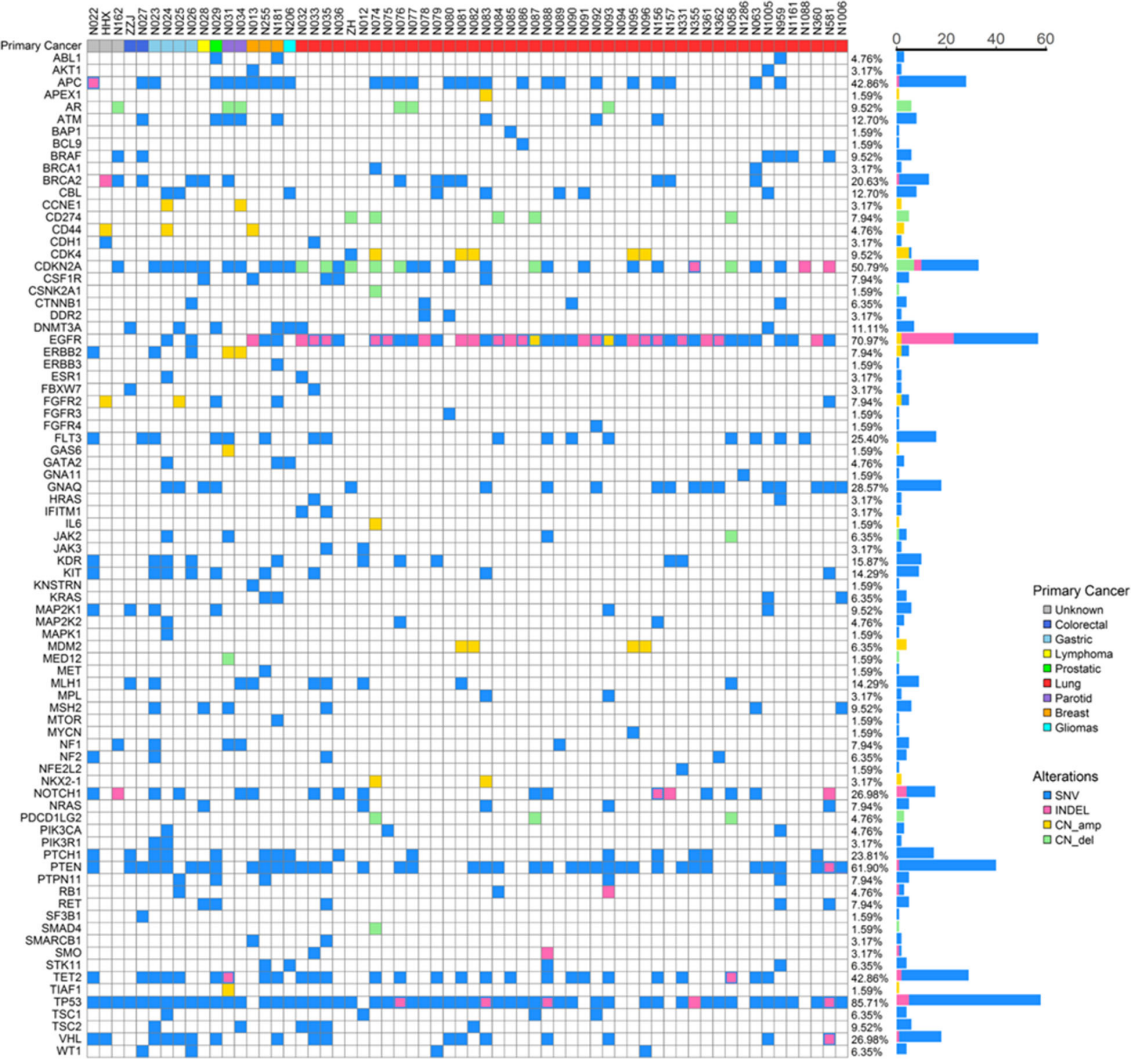
Profiling of genes mutations in the CSF samples obtained from NM patients, regardless of the primary cancer origin

**Fig. 2:**
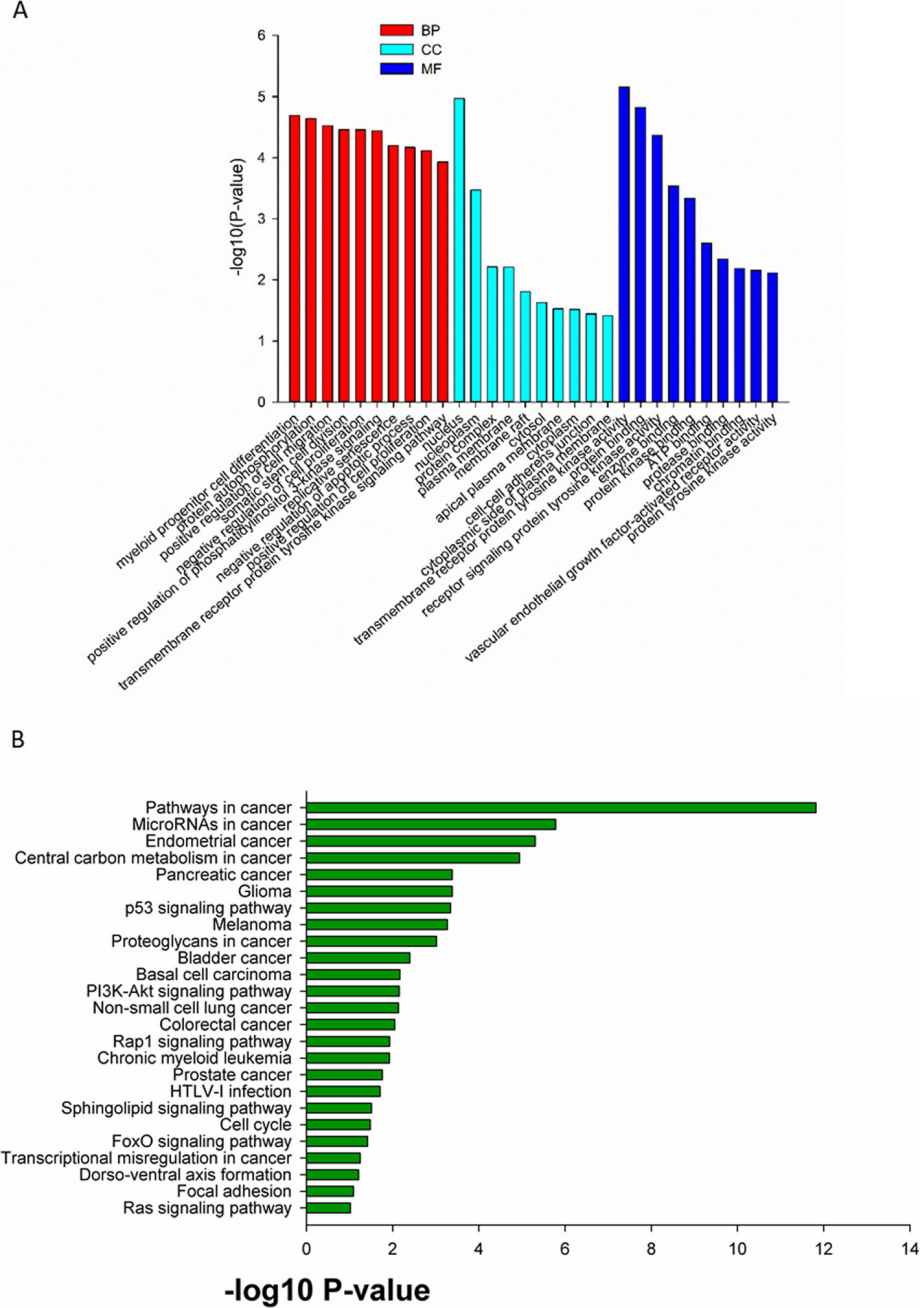
The GO analysis (**a**) and KEGG pathway analysis (**b**) of mutated genes in the CSF obtained from NM patients regardless of the primary cancer origin. BP, biological process; CC, cellular components; MF, molecular function

The 62 CSF samples were all positive for ctDNA and mutations of cancer-associated genes. Specifically, 68 (47.6%) of the 143 cancer-associated genes analyzed in the present study had mutations in at least one NM CSF sample, and 62 (100%) of the NM CSF samples carried at least one mutated gene. The most commonly mutated gene was *TP53* (54/62, 87.10%), followed by *EGFR* (44/62, 70.97%), *PTEN* (39/62, 62.90%), *CDKN2A* (32/62, 51.61%), *APC* (27/62, 43.55%), *TET2* (27/62, 43.55%), *GNAQ* (18/62, 29.03%), *NOTCH1* (17/62, 27.42%), *VHL* (17/62, 27.42%), *FLT3* (16/62, 25.81%), *PTCH1* (15/62, 24.19%), *BRCA2* (13/62, 20.97%), *KDR* (10/62, 16.13%), *KIT* (9/62, 14.52%), *MLH1* (9/62, 14.52%), *ATM* (8/62, 12.90%), *CBL* (8/62, 12.90%), and *DNMT3A* (7/62, 11.29%) (Fig. 1). Furthermore, GO (Gene Ontology) annotation and KEGG (Kyoto Encyclopedia of Genes and Genomes) pathway analyses were used to explore the potential functions of these high frequency mutated genes, and it was found that these high frequency mutated genes were rich in the PI3K-Akt signaling pathway (Fig. 2).

**Fig. 3:**
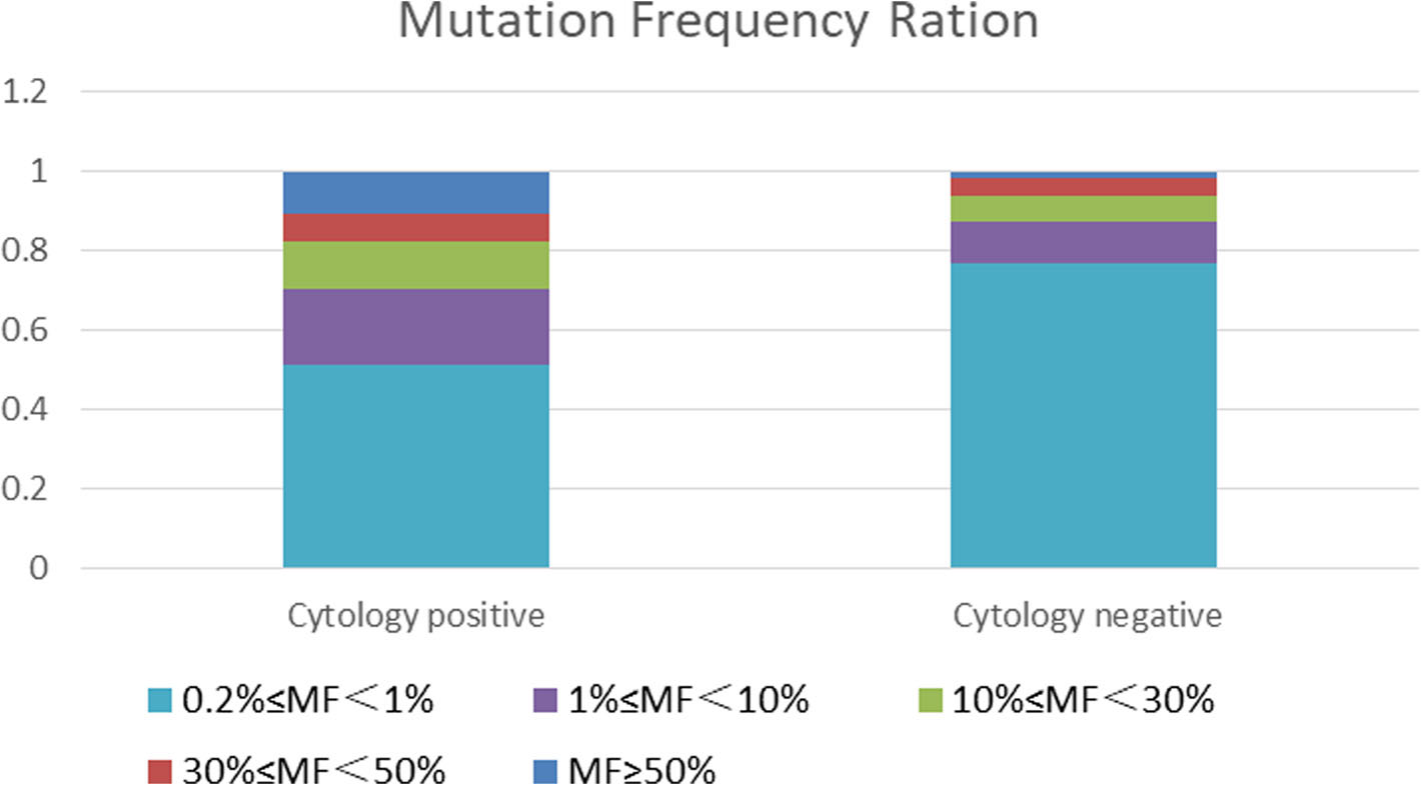
The association of variant allele frequency with detectable tumor cells in the CSF. MF, mutation frequency

Furthermore, the variant allele frequency was divided into five groups: ≥50, 30–50%, 10–30%, 1–10% and 0.2–1%, respectively. Then, the variant allele frequency was associated with the detectable tumor cells in the CSF samples, and it was found that the frequency of variant allele frequency (≥1%) was higher in the group with detectable tumor cells than that in the group without detectable tumor cells (*P* < 0.001, Fig. 3).

### Copy number variations (CNVs)

Data on the CNVs in these CSF ctDNA samples were also obtained, and it was found that high CNVs occurred in 22 of 58 NM patients. Among these 22 patients, the primary tumors were 15 lung cancers (13 lung adenocarcinoma, one squamous cell carcinoma and one unspecified), four gastric cancers, and one each of breast cancer, parotid carcinoma, and unknown primary cancer. The deletion of the *CDKN2A* copy number was the most frequent CNV that occurred in seven CSF ctDNA samples from six non-small cell lung cancers (6/22, 27.3%). In the increase in *CDK4* copy number that occurred in five lung adenocarcinomas, four of these exhibited an increase in *MDM2* copy number. In addition, an increase in *MDM2* copy number was also detected from another lung adenocarcinoma patient. Two CSF ctDNA samples had a gain of *ERBB2* (*HER2*) copy number from a parotid carcinoma patient, while an increase in *CD44* copy number was identified in three patients, in which each patient has breast cancer, gastric cancer and unknown cancer, respectively. In addition, an increased *EGFR* copy number occurred in three lung adenocarcinoma patients. Other CNVs of tumor-associated genes were detected in five patients (six positive CSF ctDNA samples) with decreased *AR* copy numbers, five patients had decreased *CD274* copy numbers, three patients each has a decreased *PDCD1LG2* copy number, two patients each has an increase in *FGFR2*, *CCNE1*, or *NKX2–1* copy numbers, respectively, and one patient had increased *TIAF1*, *GAS6*, or *IL6* copy numbers, or reduced *CSNK2A1*, *JAK2*, *MED12*, *or SMAD4* copy numbers.

### Association of gene mutations with intrathecal chemotherapy and systemic therapy

**Fig. 4:**
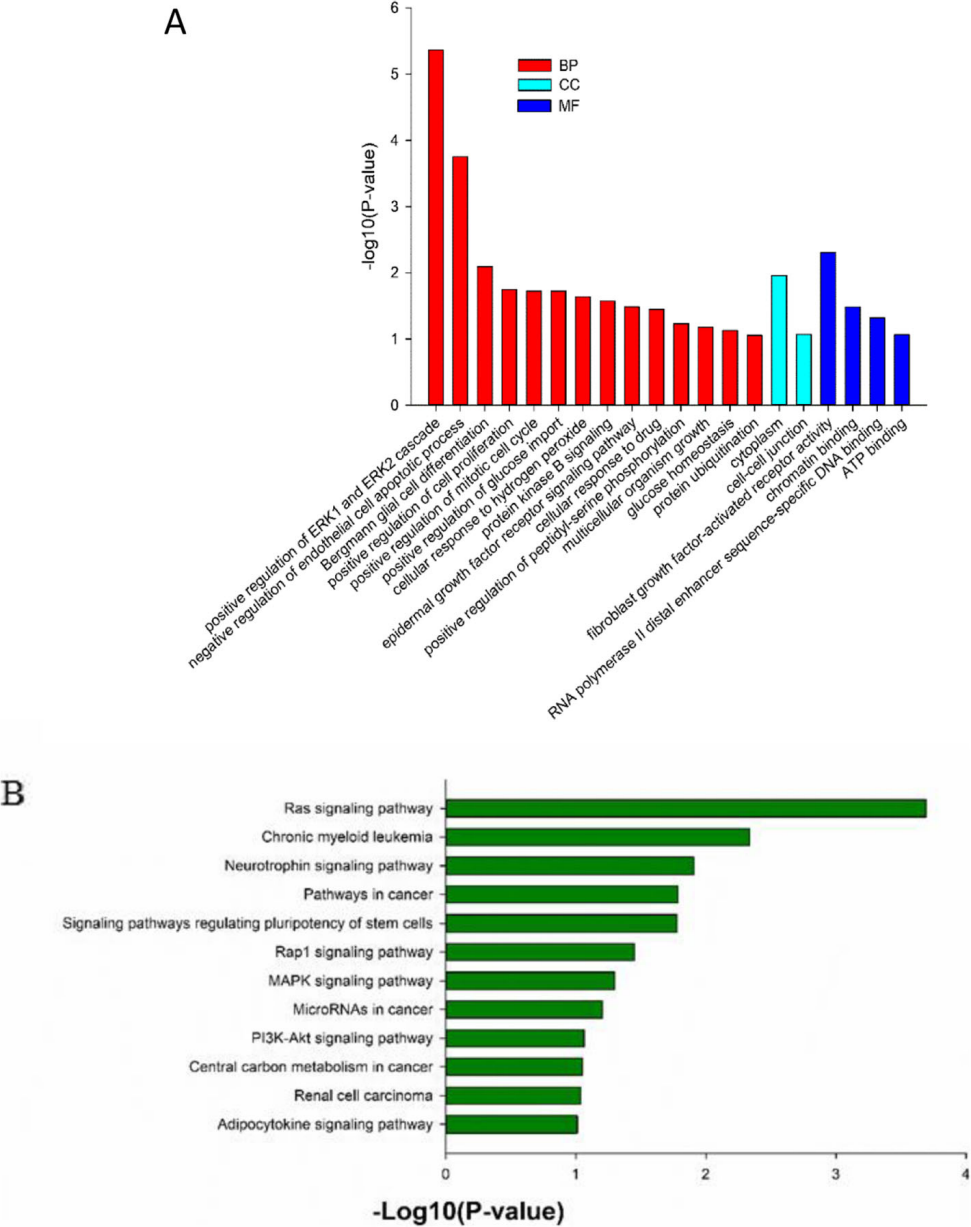
The GO analysis (**a**) and KEGG pathway analysis (**b**) of mutated genes in the CSF obtained from NM patients receiving both intrathecal chemotherapy and systemic therapy. BP, biological process; CC, cellular components; MF, molecular function.

The data on these unique mutated genes were summarized for each group of patients, followed by a gene-annotation enrichment analysis. It was found that the ERK1/2 pathway was mostly enriched by the GO analysis in patients who received both intrathecal chemotherapy and systemic therapy (Fig. 4 and Table 2).

**Table 2:** Unique mutated genes in each treatment group

Mutated genes	IC and ST (30 samples)	IC without ST (11 samples)	ST without IC (12 samples)	Neither IC nor ST (9 samples)
ERBB3	5.26%	WT	WT	WT
FGFR3	5.26%	WT	WT	WT
FGFR4	5.26%	WT	WT	WT
GAS6	5.26%	WT	WT	WT
GNA11	5.26%	WT	WT	WT
KNSTRN	5.26%	WT	WT	WT
MED12	5.26%	WT	WT	WT
MTOR	5.26%	WT	WT	WT
MYCN	5.26%	WT	WT	WT
NFE2L2	5.26%	WT	WT	WT
TIAF1	5.26%	WT	WT	WT
AKT1	10.53%	WT	WT	WT
FBXW7	10.53%	WT	WT	WT
HRAS	10.53%	WT	WT	WT
ABL1	15.79%	WT	WT	WT
BAP1	WT	12.50%	WT	WT
MAPK1	WT	12.50%	WT	WT
SF3B1	WT	12.50%	WT	WT
PIK3R1	WT	25.00%	WT	WT
CSNK2A1	WT	WT	10.00%	WT
IL6	WT	WT	10.00%	WT
MET	WT	WT	10.00%	WT
SMAD4	WT	WT	10.00%	WT
APEX1	WT	WT	WT	11.11%
BCL9	WT	WT	WT	11.11%

Abbreviations: IC, intrathecal chemotherapy; ST, systemic therapy; WT, wide type

### The association of CSF ctDNA concentration with Karnofsky performance status (KPS) score, gene mutation and CSF tumor cells

**Fig. 5:**
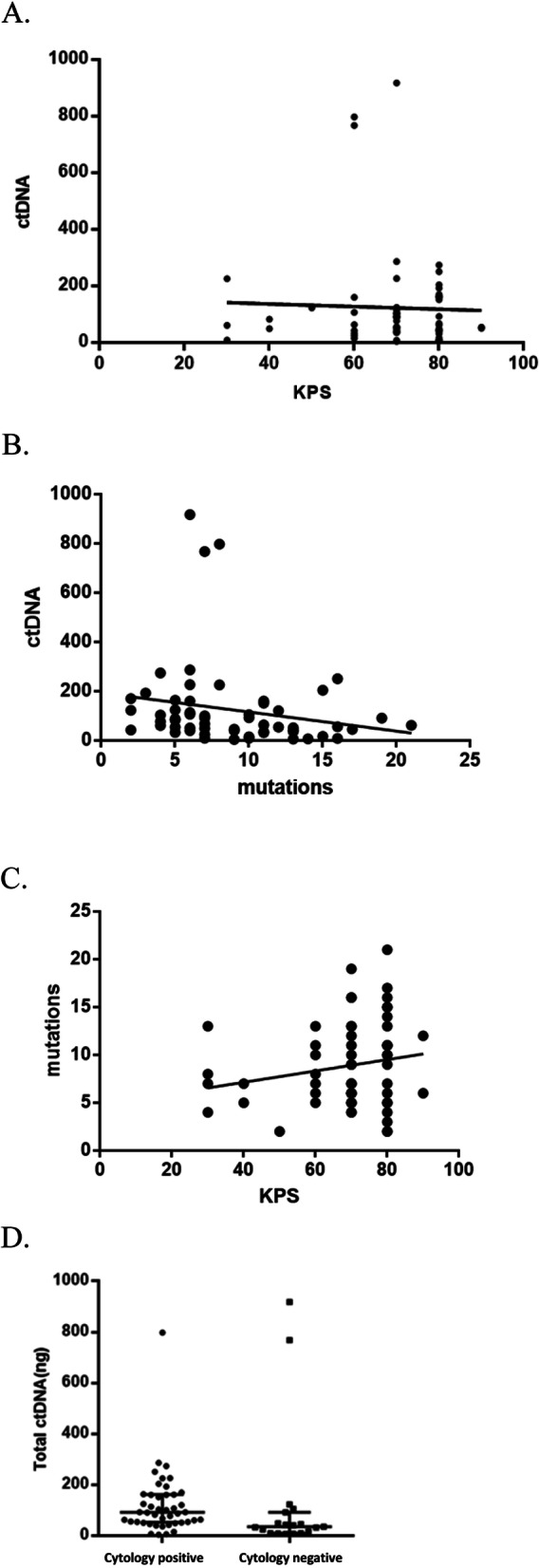
The association of CSF ctDNA concentration with the Karnofsky performance status (KPS) scores, the number of gene mutations and the presence of CSF tumor cells. (A) CSF ctDNA concentration vs. KPS (r = − 0.038, *P* = 0.768). (B) CSF ctDNA concentration vs. the number of gene mutations (r = − 0.195, *P* = 0.129). (C) The number of gene mutations vs. KPS scores (r = 0.192, *P* = 0.135). (D) CSF ctDNA concentration vs. the presence of detectable circulating tumor cells in the CSF (Z = -2.883, *P* = 0.004)

The CSF ctDNA concentration was not statistically associated with the KPS scores (r = − 0.038, *P* = 0.768; Fig. 5a) or the number of gene mutations (r = − 0.195, *P* = 0.129; Fig. 5b). Furthermore, the number of gene mutations was not associated with the KPS score (r = 0.192, *P* = 0.135; Fig. 5c). However, it was found that the CSF ctDNA concentration was associated with tumor cells in the CSF, when compared to that without circulating tumor cells (Z = -2.883, *P* = 0.004; Fig. 5d).

### Cancer-associated genes mutations in the 45 CSF samples obtained from 42 NM patients with lung cancer

A total of 45 CSF samples were collected from 42 NM patients with lung cancer, in which three CSF samples were collected from a single patient at distinct time points, while two CSF samples were collected from the other patient at distinct time points. In the subgroup analysis, it was found that CSF ctDNA was detected in all 45 CSF samples obtained from 42 lung cancer patients with NM, and gene mutations were also detected in all patients. Specifically, *EGFR* mutations occurred in 39 of 45 CSF samples (86.67%), followed by *TP53* (38/45, 84.44%), *PTEN* (27/45, 60.00%), *TET2* (18/45, 40.00%), *APC* (17/45, 37.78%), *CDKN2A* (14/45, 31.11%), *GNAQ* (14/45, 31.11%), and *NOTCH1* (11/45, 24.44%). A number of gene mutations previously reported with lung cancer were identified in CSF with NM, while *EGFR*, *TP53*, *PTEN*, *TET2*, *APC*, *CDKN2A*, *GNAQ*, *NOTCH1*, *FLT3*, *VHL*, *BRCA2*, *PTCH1*, *CBL*, *MLH1*, *BRAF*, *NRAS*, *TSC2*, *CSF1R*, *KIT*, *MAP2K1*, *MSH2*, *TSC1*, *HRAS*, *IFITM1* and *BCL9* mutations were statistically more common in the present cohort of NM, when compared to the lung cancer noted in the COSMIC database (https://cancer.sanger.ac.uk) (Table 3).

**Table 3:** The number of mutation-bearing CSF samples (total number of investigated CSF samples: 45) per cancer-associated gene (total number of investigated genes: 143)

Genes	NM (Our cohort)	Lung cancer (COSMIC database)	p-value
ABL1	1/45 (2.22%)	64/5425 (1.18%)	0.417
AKT1	1/45 (2.22%)	63/10898 (0.58%)	0.232
APC	17/45 (37.78%)	200/5979 (3.35%)	**< 0.001**
ATM	3/45 (6.67%)	288/5300 (5.43%)	0.974
BAP1	1/45 (2.22%)	51/4574 (1.11%)	0.401
BCL9	1/45 (2.22%)	0/2603 (0.00%)	**0.017**
BRAF	4/45 (8.89%)	596/26989 (2.21%)	**0.033**
BRCA1	2/45 (4.44%)	122/4786 (2.55%)	0.744
BRCA2	7/45 (15.56%)	156/4753 (3.28%)	**< 0.001**
CBL	5/45 (11.11%)	69/4871 (1.42%)	**0.001**
CDH1	1/45 (2.22%)	50/5014 (1.00%)	0.367
CDK4	1/45 (2.22%)	11/4572 (0.24%)	0.111
CDKN2A	14/45 (31.11%)	558/7621 (7.32%)	**< 0.001**
CSF1R	3/45 (6.67%)	82/4775 (1.72%)	**0.044**
CTNNB1	3/45 (6.67%)	157/6679 (2.35%)	0.161
DDR2	2/45 (4.44%)	139/6918 (2.01%)	0.231
DNMT3A	2/45 (4.44%)	104/4644 (2.24%)	0.627
EGFR	39/45 (86.67%)	26,099/98618 (26.46%)	**< 0.001**
ESR1	1/45 (2.22%)	64/4425 (1.45%)	0.484
FBXW7	1/45 (2.22%)	131/5455 (2.4%)	1.000
FGFR2	1/45 (2.22%)	64/5603 (1.14%)	0.407
FGFR3	1/45 (2.22%)	65/6466 (1.01%)	0.369
FGFR4	1/45 (2.22%)	57/4569 (1.25%)	0.436
FLT3	10/45 (22.22%)	103/5948 (1.73%)	**< 0.001**
GNA11	1/45 (2.22%)	18/4780 (0.38%)	0.163
GNAQ	14/45 (31.11%)	24/5036 (0.48%)	**< 0.001**
HRAS	2/45 (4.44%)	33/7105 (0.46%)	**0.020**
IFITM1	2/45 (4.44%)	3/2468 (0.12%)	**0.003**
JAK2	1/45 (2.22%)	102/6912 (1.48%)	0.490
JAK3	2/45 (4.44%)	88/5176 (1.7%)	0.181
KDR	5/45 (11.11%)	218/5112 (4.26%)	0.060
KIT	3/45 (6.67%)	120/6695 (1.79%)	**0.048**
KRAS	2/45 (4.44%)	6809/42506 (16.02%)	**0.034**
MAP2K1	3/45 (6.67%)	58/9328 (0.62%)	**0.003**
MAP2K2	1/45 (2.22%)	23/4529 (0.51%)	0.212
MLH1	5/45 (11.11%)	36/4969 (0.72%)	**< 0.001**
MPL	2/45 (4.44%)	39/4946 (0.79%)	0.052
MSH2	3/45 (6.67%)	54/4595 (1.18%)	**0.017**
MYCN	1/45 (2.22%)	34/4544 (0.75%)	0.293
NF1	1/45 (2.22%)	303/4814 (6.29%)	0.416
NF2	2/45 (4.44%)	51/4865 (1.05%)	0.084
NFE2L2	1/45 (2.22%)	212/5626 (3.77%)	0.881
NOTCH1	11/45 (24.44%)	197/6456 (3.05%)	**< 0.001**
NRAS	4/45 (8.89%)	128/16010 (0.8%)	**0.001**
PIK3CA	2/45 (4.44%)	719/16029 (4.49%)	1.000
PTCH1	7/45 (15.56%)	86/5107 (1.68%)	**< 0.001**
PTEN	27/45 (60.00%)	233/8576 (2.72%)	**< 0.001**
PTPN11	2/45 (4.44%)	54/5910 (0.91%)	0.066
RB1	2/45 (4.44%)	369/5651 (6.53%)	0.794
RET	3/45 (6.67%)	142/5818 (2.44%)	0.181
SMARCB1	1/45 (2.22%)	31/4968 (0.62%)	0.251
SMO	2/45 (4.44%)	72/5105 (1.41%)	0.136
STK11	2/45 (4.44%)	594/8028 (7.4%)	0.646
TET2	18/45 (40.00%)	90/4349 (2.07%)	**< 0.001**
TP53	38/45 (84.44%)	4456/11831 (37.66%)	**< 0.001**
TSC1	3/45 (6.67%)	70/4775 (1.47%)	**0.030**
TSC2	4/45 (8.89%)	125/4800 (2.6%)	**0.032**
VHL	9/45 (20.00%)	23/5332 (0.43%)	**< 0.001**
WT1	2/45 (4.44%)	62/4634 (1.34%)	0.125

The differences between the mutation rates observed in our cohort and those noted in the COSMIC database (https://cancer.sanger.ac.uk) were statistically compared. Significant *p*-values are marked with bold text

### Enriched genes and gene pathways in NM patients with lung cancer

**Fig. 6:**
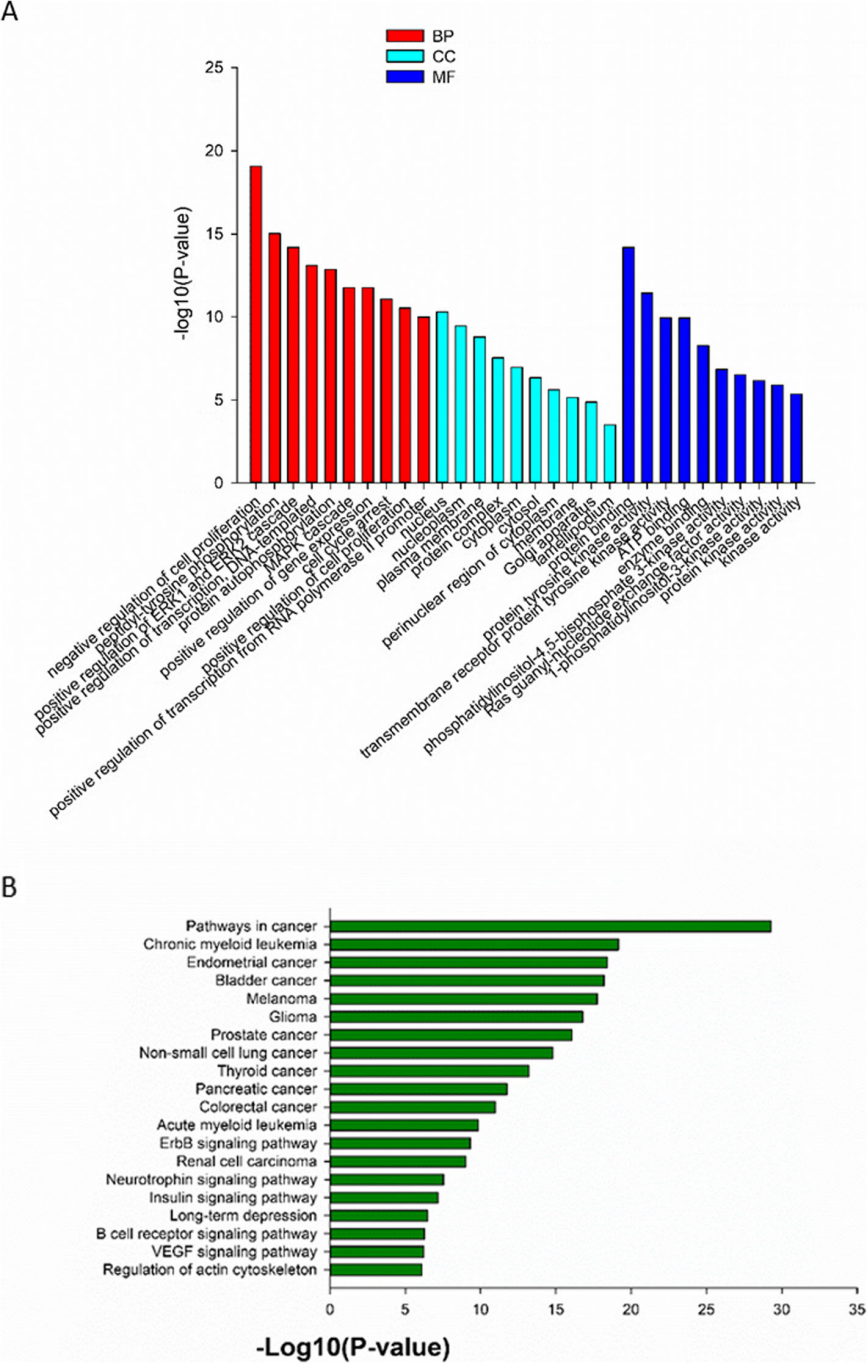
The data on the GO analysis (**a**) and KEGG pathway analysis (**b**) of mutated genes in the CSF obtained from NM patients with lung cancer

Mutated genes in the 42 NM patients with lung cancer were analyzed by GO annotation and KEGG pathway analyses. The top three GO terms were negative regulation of cell proliferation, peptidyl-tyrosine phosphorylation and positive regulation of ERK1 and ERK2 cascade. KEGG pathway analysis found these genes were associated with various biological processes, which included the general signaling pathways underlying the progression of cancer (*P* = 5.21 × 10^− 30^; *q* = 5.05 × 10^− 28^), chronic myeloid leukemia (*P* = 7.01 × 10^− 20^; *q* = 3.40 × 10^− 18^), endometrial cancer (*P* = 3.82 × 10^− 19^; *q* = 1.24 × 10^− 17^), bladder cancer (*P* = 6.39 × 10^− 19^; *q* = 1.55 × 10^− 17^), melanoma (*P* = 1.78 × 10^− 18^; *q* = 3.44 × 10^− 17^), glioma (*P* = 1.62 × 10^− 17^; *q* = 2.61 × 10^− 16^), prostate cancer (*P* = 8.66 × 10^− 17^; *q* = 1.20 × 10^− 15^), and non-small cell lung cancer (*P* = 1.59 × 10^− 15^; *q* = 1.93 × 10^− 14^), while the related signaling pathways were the ErbB signaling (*P* = 4.92 × 10^− 10^; *q* = 3.67 × 10^− 9^), VEGF signaling (*P* = 6.00 × 10^− 7^; *q* = 3.06 × 10^− 6^), MAPK signaling (*P* = 1.33 × 10^− 6^; *q* = 5.88 × 10^− 6^), p53 signaling (*P* = 4.14 × 10^− 6^; *q* = 1.61 × 10^− 5^), and m-TOR signaling (*P* = 1.00 × 10^− 5^; *q* = 3.33 × 10^− 5^) pathways (Fig. 6). Furthermore, the KEGG pathway analysis revealed that *EGFR*, *TP53*, *CDKN2A*, *CDK4*, *BRAF*, *NRAS*, *HRAS*, *JAK3*, *KRAS*, *MAP2K1*, *MAP2K2*, *PIK3CA* and *RB1* were strongly associated with non-small cell lung cancer.

**Fig. 7:**
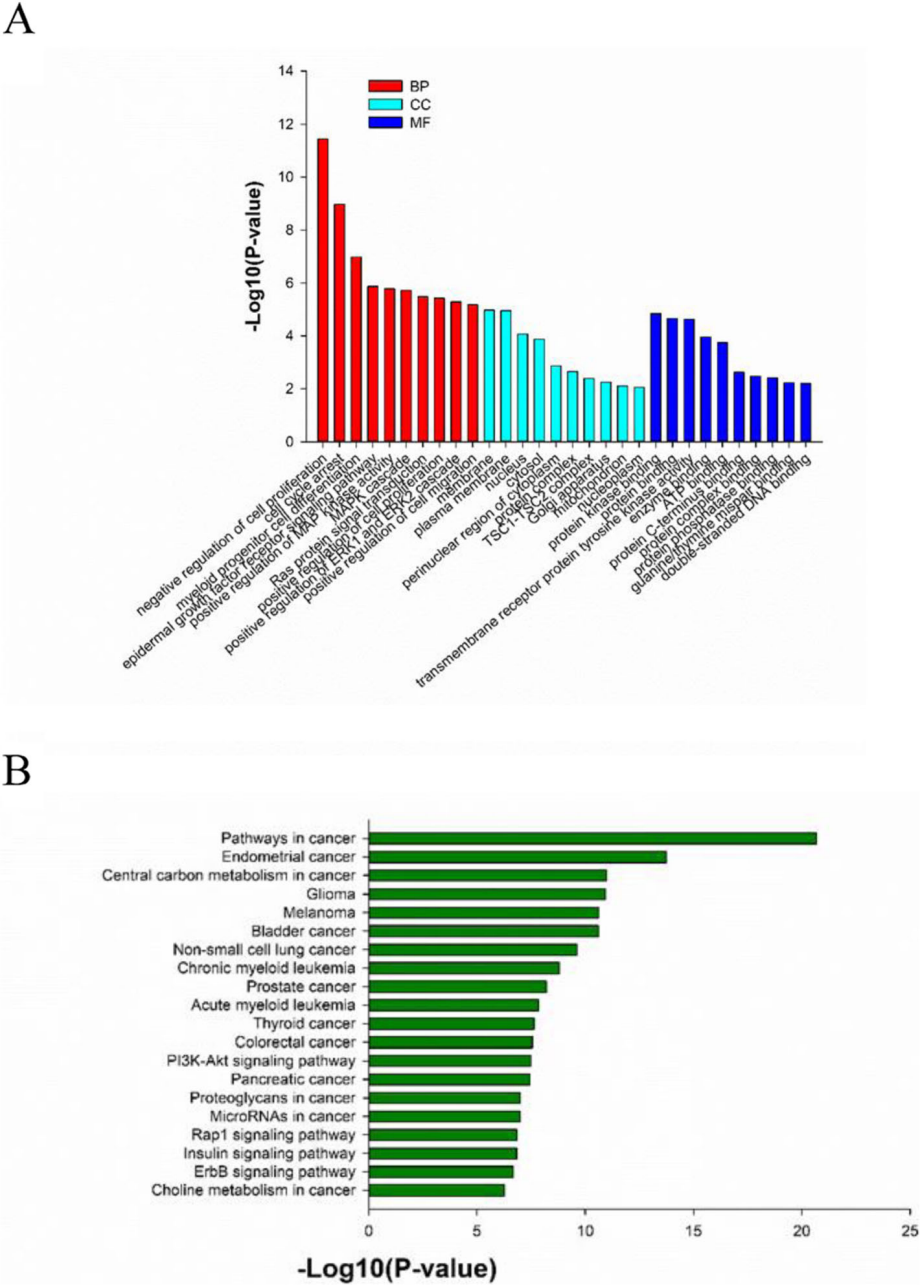
The data on the GO analysis (**a**) and KEGG pathway analysis (**b**) of significant mutated genes in the CSF obtained from NM patients with lung cancer compared to mutated genes of lung cancer in COSMIC database

A number of gene mutations were statistically more common in the present cohort of NM, when compared to the lung cancer noted in the COSMIC database, and these genes were also further analyzed by GO annotation and KEGG pathway analyses (Fig. 7).

### The association of *EGFR* mutations between lung cancer tissues and NM CSF samples

Next, *EGFR* mutations were associated between lung cancer tissues and the NM CSF samples available in 10 patients (Table 4). Specifically, CSF samples were collected from N033, N063, N077, N1088, N156, N331, N355 and N1286 during the TKI therapy, while N079 and N090 before the TKI. It was found that there were roughly the same EGFR mutations between lung adenocarcinoma tissues and CSF of nine patients, except for N1088, in which the *EGFR* mutation was undetectable in the CSF sample.

**Table 4:** *EGFR* activating mutations in primary lung cancer and NM CSF samples

CSF sample No.	Primary lung cancer	CSF
N033	EGFR 19Del	EGFR 19Del
N063	EGFR L858R	EGFR L858R, E709A, T790M
N077	EGFR L858R	EGFR L858R
N079	EGFR L858R	EGFR L858R
N090	EGFR L858R	EGFR L858R
N1088	EGFR 19Del	EGFR (−)
N156	EGFR 19Del	EGFR 19Del, T790M
N331	EGFR 19Del	EGFR 19Del
N355	EGFR L858R	EGFR L858R
N1286	EGFR L858R	EGFR L858R

### A representative case

**Fig. 8:**
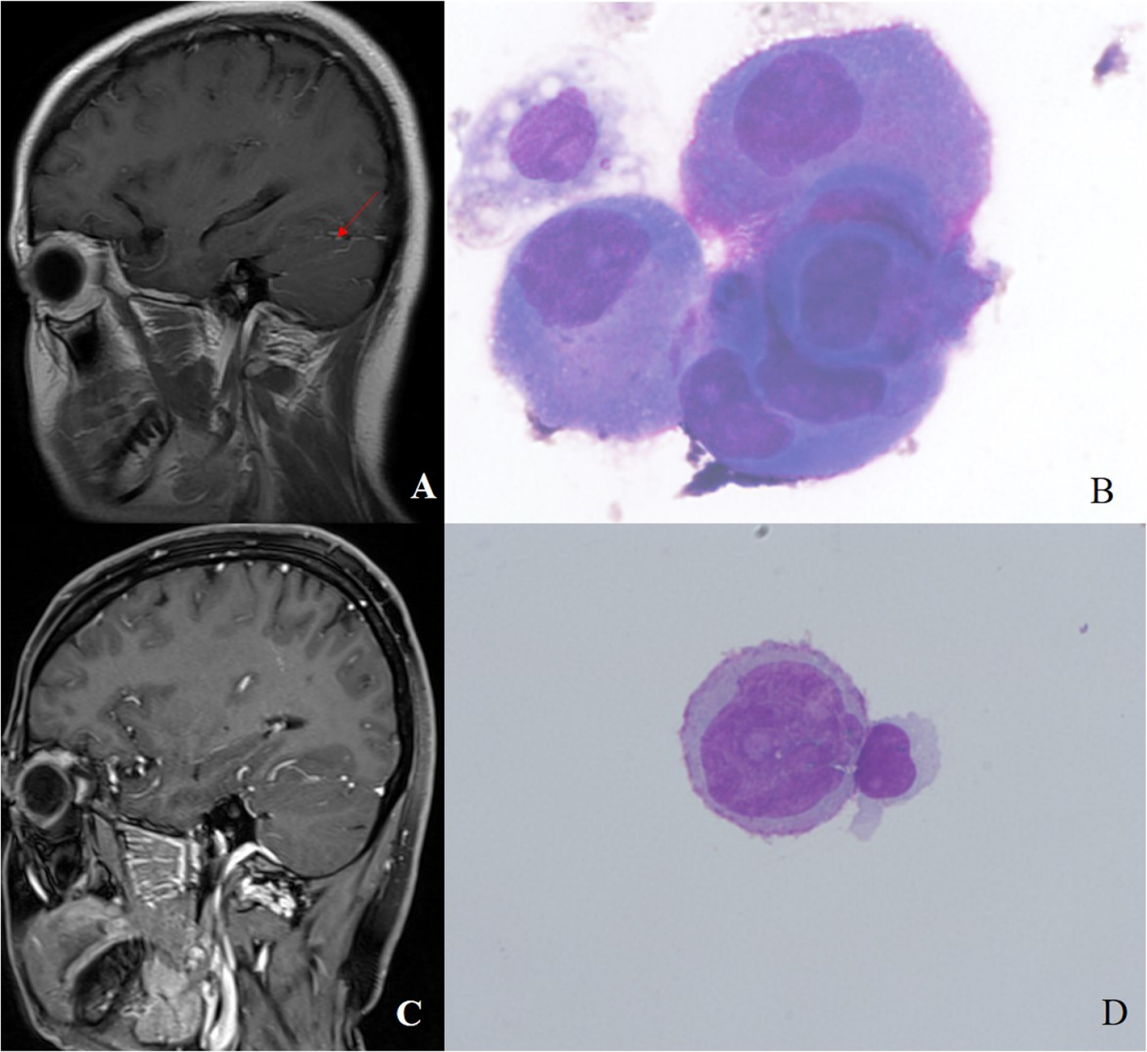
A representative case. (**a**) The head contrast enhanced MRI. This showed the linear and strip abnormal enhancement of the cerebellar sulcus (red arrow) after the patient had a headache during the icotinib therapy for the primary tumor. (**b**) The May-Gruwald-Giemsa staining of the CSF sample. The data showed the tumor cells in the CSF (× 1000). (**c**) The head contrast enhanced MRI. This showed the dramatic improvement and complete response. (**d**) The May-Gruwald-Giemsa staining. The data showed fewer tumor cells (× 1000), when compared to that in B

In the present cohort, there was a lung adenocarcinoma patient who underwent surgical lung cancer resection, and tumor tissues had an *EGFR* 19Del mutation detected by NGS. Thus, the patient orally received 125 mg of icotinib three times a day for six months and thereafter. However, the patient had a headache during the icotinib therapy for the primary tumor. The head contrast enhanced MRI showed the linear and strip abnormal enhancement of the cerebellar sulcus (Fig. 8a) after the patient’s cancer spread into the leptomeninges, and the CSF cytology examination showed tumor cells in the CSF (Fig. 8b). Furthermore, the CSF sample revealed *EGFR* 19Del and T790M mutations in the CSF ctDNA by NGS technology. Given such a situation, the patient was given 80 mg of AZD9291 once a day to replace the icotinib for 18 months, and the patients overall health condition improved. Furthermore, a complete response was confirmed by the contrast-enhanced brain MRI (Fig. 8c), CSF cytology (Fig. 8d), and undetectable *EGFR* mutations in the CSF samples. The patient has been alive for nearly 3 years since the diagnosis of NM.

## Discussion

In the present study, NM patients were enrolled for the detection of CSF ctDNA, gene mutations and copy number variations. Furthermore, the present cohort of NM patients revealed that the large majority of primary cancers was lung adenocarcinoma, and 10 patients had NM as the first clinical manifestation, although seven of these 10 patients were clarified for their primary tumor. Afterwards, 62 CSF samples were acquired from 58 NM patients, and all samples contained detectable ctDNA, indicating that detection of CSF ctDNA is a sensitive biomarker for NM patients, since the ctDNA may not originate from benign tumors and non-neoplastic conditions, according to previous studies [6, 7]. Indeed, a previous study of ctDNA in 640 patients with different cancers [15] revealed that plasma ctDNA can be detected in at least 75% of patients vs. less than 50% patients with brain tumors, such as glioma, suggesting that CSF ctDNA can be an alternative source of samples for brain tumor diagnosis, since the present data detected positive CSF ctDNA in all 62 CSF specimens. However, it may also be observed that all patients in the present study had NM, and tumor cells in NM can disseminate over the leptomeningeal surface, followed by neoplastic cell shedding into the CSF. Thus, it needs to be further determined whether the CSF could be used to detect early stage brain tumors. However, it is true that the CSF can be a best source to detect ctDNA in NM patients. Previous studies have also reported that all 26 patients [8] and three patients [6] had positive ctDNA in the CSF samples, and the present study further supports these previous studies. In addition, the present study further demonstrated that CSF ctDNA is a useful resource to analyze gene mutations, which can help medical oncologists identify primary tumors that can cause NM. It was found that the mutations of cancer-associated genes occurred in all 62 CSF ctDNA samples, with the highest frequency on *TP53* (54/62, 87.10%), *EGFR* (44/62, 70.97%), *PTEN* (39/62, 62.90%), *CDKN2A* (32/62, 51.61%), *APC* (27/62, 43.55%), and *TET2* (27/62, 43.55%). These mutated genes enriched by the KEGG pathway analysis was the PI3K-Akt signaling pathway. The ERK1/2 signaling pathway was significantly activated in NM patients who received intrathecal chemotherapy and systemic therapy, indicating that intrathecal chemotherapy and systemic therapy might induce novel gene mutations in NM patients. The present study also identified the variation of gene copy numbers in these 62 samples. In conclusion, the data obtained from the present study demonstrates the following: (1) ctDNA is detectable in all CSF samples; (2) gene mutations are detectable in all CSF samples; (3) the gene copy number varies in all CSF samples; (4) the PI3K-Akt and ERK1/2 signaling pathways are the most altered signaling pathways for these mutated genes; (5) novel gene mutations are induced by intrathecal chemotherapy and systemic therapy in NM patients; (6) lung cancer (especially lung adenocarcinoma) is the major primary tumor in the present cohort of NM patients. Future studies would investigate the usefulness of the CSF and ctDNA for the early detection of NM patients, and target these mutated genes for the therapy of NM patients or even patients with these primary tumors.

Indeed, the PI3K-Akt signaling pathway, including but is not limited to *TP53*, *EGFR*, *PTEN*, *KIT* and *KDR*, could be crucial or at least partially crucial in mediating primary cancer for meningeal metastasis. In particular, numerous isoforms and/or spliced variants of PI3Ks participate in the regulation of various cell processes, such as cell cycle progression, cell polarization, migration, survival and metabolism, as well as tumor angiogenesis [16]. Furthermore, Akt is amenable to the vast majority of PI3K-mediated responses [17], and the alterations of Akt upstream regulators, elevated Akt expression, and/or Akt activation all result in the promotion of tumor metastasis [18]. For example, activated PI3K-Akt signaling could stimulate the translocation of α-actinin-4 from the nucleus to the cytoplasm and plasma membrane, which in turn induce changes in cell morphology and motility [19]. In human carcinogenesis, the PI3K-Akt signaling pathway inhibited the expression of tumor suppressor gene E-cadherin, which led to tumor cell epithelial mesenchymal transition and metastasis [20–22]. Previous studies have revealed that the PI3K-Akt signaling pathway plays a crucial role in the progression and metastasis of lung cancer [23], ovarian cancer [18], nasopharyngeal carcinoma [24], prostate cancer [25], colorectal cancer [26], and gastric cancer [27]. The present study further supports and confirms the important role of the PI3K-Akt signaling pathway in NM patients, which is novel, and to date, there has been no report in the literature. Hence, further studies are needed to verify the importance of this signaling pathway in NM. Furthermore, in the present study, ERK1/2 signaling was found to be enriched in NM patients after intrathecal chemotherapy and systemic therapy, indicating that the change in the ERK1/2 signaling could be associated with treatment resistance. Indeed, the inhibition of the ERK1/2 signaling pathway was caused by the *Dioscorea bulbifera*-induced apoptosis in human colorectal carcinoma cells [28]. Conversely, the GABAergic signaling facilitated breast cancer metastasis through promotion of the ERK1/2-dependent phosphorylation [29]. This speculation needs to be further studied to determine whether this is associated with treatment resistance to both intrathecal chemotherapy and systemic therapy.

In addition, the present study also identified the CNVs of different genes in the CSF ctDNA samples, and the most affected ones were *CDKN2A*, *CDK4* and *MDM2*. As it is known, the deletion of tumor suppressor *CDKN2A* was associated with melanoma and pancreatic neuroendocrine tumors metastasis, and the reduced survival rate of patients [30, 31]. Indeed, during the DNA replication in cells, gene amplification could generally create a risk for gene overexpression, which could be involved in cancer initiation and progression [32]. Furthermore, a previous study revealed that *CDK4* and *MDM2* mutations occurred in melanomas and liposarcoma, while *ERBB2* mutations occurred in breast cancer, *EGFR* mutations occurred in astrocytoma, and *MYCN* mutations occurred in neuroblastoma [32]. Altered *CD44* expression was associated with the aggressive clinicopathological characteristics of various human cancers [33]. In addition, the present study revealed the EGFR and *TP53* mutations in the CSF samples of NM patients with lung cancer, in which the COSMIC database also confirmed that these were the most frequently mutated genes in the CSF of NM patients, when compared to primary lung cancer. Furthermore, a higher mutational rate was found from *EGFR*, *TP53*, *PTEN*, *TET2*, *APC*, *CDKN2A*, *GNAQ*, *NOTCH1*, *FLT3*, *VHL*, *BRCA2*, *PTCH1*, *CBL*, *MLH1*, *BRAF*, *NRAS*, *TSC2*, *CSF1R*, *KIT*, *MAP2K1*, *MSH2*, *TSC1*, *HRAS*, *IFITM1* and *BCL9* genes in NM with lung cancer, when compared to lung cancer patients without NM. A previous study revealed that EGFR mutations predisposed to the leptomeningeal metastases of *EGFR*-mutant non-small-cell lung cancer [34], and the present study further supports this notion. In spite of the relatively small sample size, the rate of the above mutated genes was much higher than what was reported in patients with lung adenocarcinoma from the COSMIC database, implicating that these genes mutations and the alteration of the signaling pathways are involved in and have become risk factors for NM.

After associating these *EGFR* activating mutations between primary lung cancer and NM, the present data shows that the *EGFR* activating mutations in the CSF samples were roughly consistent with those of primary lung cancer. For example, the T790M in two CSF samples were sequentially collected during the TKI therapy. Furthermore, according to the gene set enrichment analysis, it was found that the ErbB, VEGF, MAPK, and m-TOR signaling pathways were significantly enriched in the 45 CSF samples obtained from NM patients with lung cancer, suggesting that the alterations of these signaling pathways might promote NM in lung cancer patients. In particular, the ErbB signaling pathway can regulate cell proliferation, migration, differentiation and apoptosis through the crosstalk with the PI3K-Akt, MAPK, or other signaling pathways [35].

Lastly, the representative patient in the present study provided a unique showcase. The genetic profiling of the CSF ctDNA clearly reflected the dynamic changes in these identified driver genes and treatment responses. That is, this patient was detected to have the *EGFR* 19Del mutation in the CSF sample, and thereby received icotinib. Thereafter, the CSF sample revealed the *EGFR* 19Del and T790M mutations. Hence, the treatment was switched to AZD9291, which is a third generation *EGFR* TKI agent. A high response rate was exhibited by patients with tumors harboring the *EGFR* T790M mutation, as well as a high capacity to penetrate into the CSF by crossing the blood-brain barrier [36, 37]. The NGS allowed the physician to modify treatment option, in order to help the patient archive complete remission. As it is known, the *EGFR* T790M mutation mediates the acquired resistance to *EGFR* TKI [38, 39], and a previous study reported that AZD9291 was designated as a powerful agent capable of overcoming the acquired *EGFR* T790M resistance mutation [40].

However, the present study does have some limitations. For example, the cohort has a relatively small number of patients, and the different treatment options could not be separated to analyze the association of gene mutation, and the changes in gene copy number with treatment responses in these patients. Thus, future studies with a larger sample size from multiple institutions could help to solve these issues. Though we have found that mutated genes detected in CSF are enriched in the PI3K-Akt and ERK1/2 signaling pathways, it is hard for us to explain the detected mutations are gain of function mutations or loss of function mutations in such pathways. Thus, we will perform the functional analysis in the following studies. Due to the target amplicon sequencing, we could not address the whole genome alterations and yield the comprehensive landscape of NM. So there are any other area we will focus on to make more depth analyses in the future studies.

## Conclusions

This study identified gene mutations in all CSF ctDNA samples, indicating that these mutated genes may be acted as a kind of biomarker for diagnosis of NM, and these mutated genes may affect meningeal metastasis through PI3K-Akt signaling pathway.

## Supplementary information

**Additional file 1.** Table 1 The gene list of SV-OCP143-ctDNA panel

## Data Availability

The data that support the findings of this study are available from San Valley Biotechnology Incorporate but restrictions apply to the availability of these data, which were used under license for the current study, and so are not publicly available. Data are however available from the authors upon reasonable request.

## Abbreviations

CNVs: Copy number variations
CSF: Cerebrospinal fluid
ctDNA: Circulating tumor DNA
NM: Neoplastic meningitis
EGFR: Epidermal growth factor receptor
NSCLC: Non-small cell lung cancer
MRI: Magnetic resonance imaging
CT: Computed tomography
