# Supplementary material for: Detection of genes mutations in cerebrospinal fluid circulating tumor DNA from neoplastic meningitis patients using next generation sequencing
Source: BMC Cancer. 2020 Jul 25;20:690. doi: 10.1186/s12885-020-07172-x (PMC7382072; doi:10.1186/s12885-020-07172-x)
Supplement: Supplementary file 1 — Additional file 1. Table 1 The gene list of SV-OCP143-ctDNA panel [file 12885_2020_7172_MOESM1_ESM.docx]

Table 1 The gene list of SV-OCP143-ctDNA panel

| Hotspot Genes | | | Full-Length Genes | Copy Number Genes | |
| --- | --- | --- | --- | --- | --- |
| ALK | HNF1A | RAC1 | BAP1 | ACVRL1 | IGF1R |
| AR | HRAS | RAF1 | BRCA1 | AKT1 | IL6 |
| ARAF | IDH1 | RET | BRCA2 | APEX1 | KIT |
| BRAF | IDH2 | RHEB | CDKN2A | AR | KRAS |
| BTK | JAK1 | RHOA | FBXW7 | ATP11B | MCL1 |
| CBL | JAK2 | SF3B1 | MSH2 | BCL2L1 | MDM2 |
| CDK4 | JAK3 | SMO | NF1 | BCL9 | MDM4 |
| CHEK2 | KDR | SPOP | NF2 | BIRC2 | MET |
| CSF1R | KIT | SRC | NOTCH1 | BIRC3 | MYC |
| CTNNB1 | KNSTRN | STAT3 | PIK3R1 | CCND1 | MYCL |
| DDR2 | KRAS | U2AF1 | PTCH1 | CCNE1 | MYCN |
| EGFR | MAGOH | XPO1 | PTEN | CD274 | MYO18A |
| ERBB2 | MAP2K1 | ABL1 | RB1 | CD44 | NKX2-1 |
| ERBB3 | MAP2K2 | AKT1 | SMARCB1 | CDK4 | NKX2-8 |
| ERBB4 | MAPK1 | DNMT3A | STK11 | CDK6 | PDCD1LG2 |
| ESR1 | MAX | GNAS | TP53 | CSNK2A1 | PDGFRA |
| EZH2 | MED12 | IFITM1 | TSC1 | DCUN1D1 | PIK3CA |
| FGFR1 | MET | IFITM3 | TSC2 | EGFR | PNP |
| FGFR2 | MTOR | MLH1 | APC | ERBB2 | PPARG |
| FGFR3 | MYD88 | MPL | ATM | FGFR1 | RPS6KB1 |
| FLT3 | NFE2L2 | NPM1 | CDH1 | FGFR2 | SOX2 |
| FOXL2 | NRAS | PAX5 | GATA3 | TERT | FGFR3 |
| GATA2 | PDGFRA | PTPN11 | TET2 | TIAF1 | FGFR4 |
| GNA11 | PIK3CA |  | VHL | ZNF217 | FLT3 |
| GNAQ | PPP2R1A |  | WT1 |  | GAS6 |
|  |  |  | SMAD4 |  |  |
